# At your service: supportiveness of servant leadership, communication frequency and communication channel fostering job satisfaction across generations

**DOI:** 10.3389/fpsyg.2023.1183203

**Published:** 2023-08-31

**Authors:** Martine J. H. Coun, Melanie De Ruiter, Pascale Peters

**Affiliations:** ^1^Management Sciences, Open Universiteit, Heerlen, Netherlands; ^2^Center for Strategy, Organization and Leadership, Nyenrode Business Universiteit, Breukelen, Netherlands; ^3^Organization, Leadership and Management, Inland School of Business and Social Sciences, Lillehammer, Norway

**Keywords:** job satisfaction, servant leadership, communication frequency and channels, selfdetermination theory, media richness, synchronicity, remote working, generations

## Abstract

**Introduction:**

The present study contributes to the conversations on the role of ‘autonomy supportive’ factors in employee wellbeing in remote work contexts by examining the relationships between servant leadership, communication frequency – overall and *via* synchronous (i.e., individual video-calls, individual telephone calls) and asynchronous communication channels (i.e., e-mail messages, and WhatsApp) – on the one hand, and job satisfaction, on the other, and the moderating role of generation (Baby Boomers and Gen X versus Gen Y) in these relationships.

**Method:**

Building on self-determination theory, incorporating insights from servant leadership, telework, and media richness and synchronicity literatures, we developed hypotheses that were tested *via* multilevel analysis (273 employees nested in 89 managers).

**Results:**

In line with expectations, servant leadership had a positive relationship with job satisfaction. Total communication frequency, however, was not related to job satisfaction. Further analyses per communication channel showed that only level 2 e-mail communication frequency was positively related to job satisfaction. In contrast to expectations, the relationships studied were not moderated by generation.

**Discussion:**

We concluded that, for all generations, both servant leadership and frequent (e-mail) communication can be regarded as ‘autonomy supportive’ factors in employee wellbeing. Paradoxically, whereas servant leadership, considered as a human-centric leadership style, suggests close trust-based employment relationships, employees valued frequent asynchronous communication (*via* e-mail). Having access to information and knowledge when needed may satisfy employees’ need for autonomy (and perhaps for flexibility to engage in work and non-work activities). The insights gained in our study can inform organizations, managers, and employees, particularly in future remote work contexts.

## Introduction

Since the COVID-19 pandemic, many employees have started looking for new job opportunities as they are no longer satisfied with their current job (PwC’s Global Workforce Hopes and Fears Survey, 2022). Related to this, Gratton (2023) posits that the shared experience of the COVID-19 pandemic and the lockdowns in which people had to work from home more intensively than ever before has made them question what they find important in work and the rest of life. In view of this, Gratton (2023) advocated organizations to offer employees more say about how, when, and where to work, as this allows them to craft their working lives and make new connections, both within and outside work, which she links to job satisfaction.

Job satisfaction - as the extent to which people like (satisfaction) or dislike (dissatisfaction) their job (Spector, 1997) - is an indicator of employee well-being. According to Locke (1976), job satisfaction reflects “a positive emotional or pleasurable state resulting from the appraisal of one’s job or job experience” (p. 1304) and has been related to work contexts that are ‘autonomy supportive’ (Gagne, 2003; Zhang et al., 2023). This implies that people are given choice, encouraged to take initiative, experience relatedness, and feel supported to develop and use their competences (Deci et al., 2001).

Traditionally, the concept of job satisfaction has been discussed in light of social interaction and meaningful connections at work, which reflect contextual supportiveness by offering opportunities for autonomous working, information sharing, feedback, dealing with others, and friendship which, according to the self-determination theory (SDT; Deci and Ryan, 2000), can enhance the satisfaction of employees’ basic psychological needs (i.e., the need for autonomy, relatedness, and competence), autonomous motivation, performance satisfaction, and employee well-being (Gagne, 2003; Fonner and Roloff, 2010).

More specifically, job satisfaction has been related to the attention that people receive from others, such as their managers (Belias and Koustelios, 2014). Since the COVID-19 pandemic, the importance of human-centric leadership may even have increased, as employees who were forced to work from home during the pandemic needed guidance and coaching in disrupted work-family contexts (Contreras et al., 2020), which stresses the importance of establishing mutual trust in work relationships (Ahern and Loh, 2020) that is needed to enhance and sustain employees’ self-efficacy (Sweet et al., 2012).

Servant leadership, as an ‘autonomy supportive’ contextual factor that can enhance need satisfaction (Deci and Ryan, 2000; Gagne, 2003), is an important human-centric leadership approach (Van Dierendonck, 2011; Bardy, 2018; Fernandez and Shaw, 2020) that emphasizes the attentiveness of leaders to employees’ basic psychological needs and how they empathize with them (Greenleaf, 1977). Moreover, servant leaders are considered community builders who provide opportunities for employees to interact with each other. Their emotional intelligence and stability can provide the trust needed in employment relationships (Eva et al., 2019). Because of this, servant leadership may lead to positive emotional responses that affect employees’ evaluations of the (financial and non-financial) rewards of their job, such as job autonomy, flexibility, social contacts, personal development and growth, and career opportunities, which is reflected in higher job satisfaction (Aziri, 2011; Alegre et al., 2016).

In a similar vein, it can be argued that communication frequency with the supervisor can be viewed as an ‘autonomy supportive’ contextual factor that plays a role in employees’ job satisfaction. Through more frequent social interaction and meaningful connections, SDT and self-efficacy theory (Bandura, 1977) suggest that leaders can satisfy employees’ basic psychological needs which enhances their self-efficacy and autonomous motivation (Sweet et al., 2012), resulting in job satisfaction (Gagne, 2003).

Traditionally, face-to-face interaction has been considered the most salient way of interacting, as it is synchronous and enables the exchange of multiple and rich cues, allowing employees to use all their human senses, which can lead to higher levels of job satisfaction (Fonner and Roloff, 2010). In the context of remote working, however, face-to-face communication is often partly or completely replaced by communication *via* information and communication technologies (ICT), such as individual video-calls (e.g., *via* Teams, Skype, or Zoom), individual telephone calls, e-mail messages, and WhatsApp messages, which makes it more challenging for leaders to interact with their followers (Erickson, 2021). More specifically, according to media richness theory (MRT; Lengel and Daft, 1988) and media synchronicity theory (MST; Dennis and Valacich, 1999), IT-mediated communication is less ‘channel rich’ and often asynchronous compared to face-to-face communication, which hinders direct feedback and the transfer of business and personal information, signals, and personal attention (Martins et al., 2004; Lee et al., 2005; Smith et al., 2018). Even though the quality of digital communication nowadays comes close to that of face-to-face communication (Nguyen et al., 2020), making use of IT-mediated communication channels may be less effective, particularly when it comes to providing complex and ambiguous information and feedback and to signalling that employees are valued. Therefore, communication has become a constant leadership concern, not only regarding team effectiveness (Tigre et al., 2022), but also regarding job satisfaction (Paksoy et al., 2017).

Finally, the generation literature suggests that the importance of servant leadership and communication frequency and quality (i.e., type of communication channel) for job satisfaction may differ across generations. A generation, often called a cohort, refers to “an identifiable group that shares birth years, age, location, and significant life events at critical developmental stages” (Kupperschmidt, 2000, p. 66) and who have experienced similar social, historical, and life events (Mannheim, 1972). This categorization may be important to consider, particularly when it comes to a comparison of groups that have been socialized with very different technologies and lifestyles (Nicholas, 2009). Generally, four generations can be distinguished in the workplace: Baby Boomers (born 1946–1965), Gen (eration) X (born 1966–1980), Gen Y or Millennials (born 1981–2000), and Gen Z (born 2001+). The focus in this paper is on the older generations (in this study: the Baby Boomers and Gen X) versus the younger generations (in this study: Gen Y), as these are dominant and distinct groups in today’s workforce (Hart, 2006; Taylor, 2018). More specifically, particularly Gen Y may value personal support (Anderson et al., 2017) characterizing servant leadership (Barbuto and Gottfredson, 2016). Moreover, Gen Y may be more computer literate and technology ready than Baby Boomers and Gen X and, therefore, more prepared to work in remote work contexts (Pearson et al., 2010; Anderson et al., 2017; Camp et al., 2022). Although MRT (Lengel and Daft, 1988) considers e-mail and WhatsApp communication to be leaner communication channels than face-to-face communication, recent literature (Ishii et al., 2019) suggests that perceptions of media richness may differ across generations, as these much depend on the experience people have with using information and communication channels, such as text-based communication (e.g., e-mail and WhatsApp). Since technology plays an important role in remote work contexts, the generation lens can be interesting to further explore (Heuss et al., 2022).

Our study aims to contribute to the conversations on the role of ‘autonomy supportive’ factors in employee wellbeing in remote work contexts by examining the relationships between servant leadership, communication frequency with supervisor — overall and *via* synchronous (i.e., video-calls, telephone calls) and asynchronous communication channels (i.e., e-mail messages, and WhatsApp) — on the one hand, and job satisfaction, on the other, and the moderating role of generation (Baby Boomers and Gen X versus Gen Y) in these relationships.

The intended contribution of our study is threefold. First, our study extends the literature on employee wellbeing in remote work contexts by examining how both leadership style and communication frequency with the supervisor (overall and *via* synchronous and asynchronous communication channels) — as indicators of work conditions that according to SDT (Deci et al., 2017) can support employee autonomy— may foster employee wellbeing in terms of job satisfaction (Inceoglu et al., 2018). Second, we contribute to the conversation on the changing role of leadership by examining the extent to which servant leadership relates to job satisfaction, which may be particularly important for younger generations in remote work contexts (Fernandez and Shaw, 2020; Banks et al., 2022; Tigre et al., 2022). Third, we contribute to the literature on media richness by examining communication frequency, also considering the communication channel, as factor in employee wellbeing in remote work contexts (Lengel and Daft, 1988; Ishii et al., 2019). More specifically, employing a generation lens (Heuss et al., 2022), we examine how the frequent use of synchronous and asynchronous communication channels, can impact job satisfaction differently for older versus younger generations.

## Theory and hypotheses

### Servant leadership and job satisfaction

In present-day volatile and remote work contexts, directive leadership may only have short-term effects (Stoker et al., 2022). Although directive leadership can help structure the work of employees by setting clear goals and giving feedback when the work is not satisfactory, it does not stimulate employees and teams to act autonomously, to collaborate with peers inside and outside the organization, and to develop their skills and competences (Donia et al., 2016), which relates to the three basic human needs distinguished by Deci and Ryan (2000). A more appropriate leadership style to satisfy employees’ psychological needs is servant leadership (de Sousa and van Dierendonck, 2014). Servant leadership was first introduced by Greenleaf (1977), stressing leaders’ motivation to serve others and to make sure that their needs are taken care of above their own. Since then, the concept of servant leadership has been presented as a multidimensional construct (Spears, 2010; Liden et al., 2014). A seminal multidimensional conceptualisation by Van Dierendonck (2011) illustrates that servant leaders empower and develop employees, display humility, are authentic, embrace employees for what defines them, provide direction, and show stewardship. In consecutive work, Van Dierendonck et al. (2017) examined which central servant leadership dimensions were supported and solid across eight different countries. We draw from this cross-culturally validated five-dimensional conceptualization (Van Dierendonck et al., 2017) in the present study, and consider the following five servant leadership dimensions: empowerment, humility, authenticity, standing back, and stewardship.

The first dimension, *empowerment*, has two facets (Van Dierendonck et al., 2017). It focuses on enhancing people’s autonomous motivation by providing autonomy, responsibility and decision-making influence. Moreover, it has a developmental aspect which focuses on enabling employees to develop themselves. More specifically, servant leadership aims to promote a proactive attitude and self-confidence among followers (Van Dierendonck, 2011) by showing that they are valued and that their learning potential is recognized, which encourages employee development (Laub, 1999).

The second dimension, *humility,* refers to leaders’ ability to assess the true value of one’s own achievements and talents (Patterson, 2003), and acknowledge that they themselves are not flawless but also capable of making mistakes (Van Dierendonck and Nuijten, 2011).

The third dimension, *authenticity*, reflects the expression of oneself that corresponds to one’s inner thoughts and feelings. It is about being true to oneself, expressing feelings and thoughts, intentions, and commitment (Van Dierendonck and Nuijten, 2011). This leadership behavior is displayed by doing what is promised, visibility within the organization, honesty, and vulnerability (Van Dierendonck, 2011).

The fourth dimension, *standing back,* relates to a leader’s lack of pretension when withdrawing from a task that has been successfully completed (Van Dierendonck and Nuijten, 2011). Servant leaders can put other people’s interests first and offer essential support when needed (Van Dierendonck, 2011).

The fifth dimension, *stewardship*, refers to the leader’s willingness to take responsibility for the actions and performance of the team, act as a role model, and encourage others to act for the team. This characteristic is related to social responsibility, loyalty, and teamwork (Van Dierendonck, 2011), which taps into employees’ need for belongingness.

Servant leadership aims to promote job satisfaction by creating a positive work atmosphere (Eva et al., 2019). In line with this, Chan and Mak (2014) reported that servant leaders strive for high quality relationships with their employees and are eager to support and encourage them, which can enhance their need satisfaction, autonomous motivation and, ultimately, job satisfaction. Also, Jenkins and Stewart (2010) claimed that when servant leaders increase trust between themselves and their employees, employees feel valued in their jobs and gain intrinsic benefits from their work, which can impact job satisfaction. Recently, a systematic literature review by Langhof and Güldenberg (2020) revealed empirical evidence for a positive relationship between servant leadership and job satisfaction, albeit mostly in non-remote work contexts. However, particularly in times of need and crisis, such as the COVID-19 pandemic, employees are expected to experience higher levels of autonomous motivation and job satisfaction when they perceive that their organization provides support and shows concern for their wellbeing (Eisenberger et al., 2001). Therefore, we propose the following:

*Hypothesis 1*: Perceived servant leadership is positively related to job satisfaction.

### Communication frequency and job satisfaction

Communication between leaders and followers can be conceived as important for fulfilling employees’ basic psychological needs, and hence, can increase autonomous motivation and job satisfaction. Communication allows to exchange greater levels of information that help to work autonomously, to prevent professional isolation (Marshall et al., 2007) and build and maintain high-quality employment relationships, and to gain information that help employees to develop professionally (Fonner and Roloff, 2010; Stevens, 2020), which may point to the importance of communication frequency (Kacmar et al., 2003). In remote work contexts, frequent communication may have become even more important for building trust (Erickson, 2021). Missing out social face-to-face interactions and reduced interactions with supervisors and colleagues can run parallel with decreased job satisfaction among remote workers (Cooper and Kurland, 2002; Golden and Viega, 2005). In line with this, Staples (2001) found that communication frequency between manager and employees ensured a higher level of interpersonal trust and, therefore, higher levels of job satisfaction among remote workers relative to non-remote workers.

Contrary to these findings, however, Fonner and Roloff (2010) found that less frequent information exchange among high-intensity teleworkers, relative to office-based workers, related to higher job satisfaction, which could be attributed to lower stress from meetings and interruptions, and, consequently, less work-life conflict. In their view, teleworkers also perceived less office politics, which enhanced job satisfaction. Although some authors indicated that high telework-intensities (i.e., more than 15.1 h per week) may plateau the positive telework-job satisfaction relationship, suggesting a curvilinear relationship (Golden and Viega, 2005), the findings by Fonner and Roloff (2010) indicated that high-intensity teleworkers were more satisfied than collocated office workers. Strikingly, their results suggest that less interaction with others, indicating more job autonomy and independence, satisfying employees’ need for autonomy, can be beneficial. This chimes with the findings by Leonardi and Barley (2010) who found that teleworkers strategically dealt with the expectation of constant connectivity in remote work contexts by reducing their interaction intensity to focus on their work. In addition, others argued that the frequent use of digital communication can have a negative effect on employees due to too many interruptions and unpredictability of digital communication channels (Ter Hoeven et al., 2016).

In the context of the COVID-19 pandemic, however, communication may have been especially important given the uncertainty that this period entailed. Hence, particularly during the COVID-19 pandemic, leader-follower communication frequency can be seen as a proxy for perceived employee support and dealing with stress in times of crisis, which may enable employees’ need satisfaction and, subsequently, positively impact job satisfaction (Rhoades and Eisenberger, 2002). Therefore, we propose the following:

*Hypothesis 2*: Perceived communication frequency with the supervisor is positively related to job satisfaction.

### Frequency of use of communication channel and job satisfaction

In remote work contexts, also communication quality, which may depend on the communication channels used, might impact job satisfaction. According to the MRT (Daft and Lengel, 1986), the quality of communication channels (media) can be classified based on their ‘media richness’ and the equivocality of tasks. Communication media vary in their degree of “richness,” or the “ability of information to change understanding within a time interval” (Daft and Lengel, 1986, p. 561) and the extent to which the ambiguity of a message can be reduced (Daft et al., 1987). More specifically, media richness can be assessed based on four characteristics: (1) capacity of immediate feedback, (2) ability to use multiple cues, (3) personal focus provided, and (4) language variety. Based on these criteria, the richness of communication channels rank ‘face-to-face’ communication as the richest, followed by telephone, written documents, and messages, including e-mails. As new electronic communication media, such as individual video-call (e.g., *via* Teams, Skype, or Zoom) and WhatsApp, are developing rapidly, also MST (Dennis and Valacich, 1999) can be helpful to classify channels regarding these channels’ media richness. In fact, MST focuses on the same characteristics as MRT but incorporates the capability of achieving synchronicity in communication. This will be elaborated below.

*E-mail* is a text-only communication tool which runs asynchronous and has low channel richness (Lengel and Daft, 1988). Although the use of this channel lacks personalization and is limited in conveying signals, it has proven its effectiveness and mainly offers the advantage of continuity in conversations in remote work contexts (Smith et al., 2018).

Like e-mail, *instant messaging* (e.g., *WhatsApp*) is a form of textual computer-mediated communication but it allows users to communicate more synchronously when others are available than e-mail (Smith et al., 2018). These tools allow for rapid feedback and are often seen as a supplemental and informal form of communication that has gained importance in work contexts (Darics, 2020; Omar et al., 2020).

*Telephone* communication runs synchronously and allows for a greater direct exchange of (social) information. Consequently, it is considered more channel rich than e-mail. However, a disadvantage is that both parties must be available at the same time and that it does not allow messages with continued interactivity (Smith et al., 2018).

*Video conferencing* comes closest to face-to-face interactions and has a high channel richness. In high-intensity remote work contexts, this communication channel is considered to have the capacity to compensate for the absence of face-to-face interactions.

Following MRT and MST, when the communication between leaders and followers is complex and the level of ambiguity of the message content is high, face-to-face communication would be most effective (Boell et al., 2016). For a lower level of ambiguity of the message, however, a leaner medium, such as telephone or e-mail, may be more effective (Daft and Lengel, 1986). In line with this, research demonstrated that richer communication channels are more often used to communicate complex information, whilst leaner communication channels are more suitable for simple information (Matarazzo and Sellen, 2000). An empirical study by Peltokorpi (2015), for example, pointed out that, despite the many advantages associated with IT-mediated communication, face-to-face communication remained the standard for transferring tacit knowledge, whereas for communicating more explicit knowledge, such as data, lean media were shown to be more appropriate.

Although communication channels, such as e-mail, instant messaging (WhatsApp), telephone, and video calls may be more appealing and suitable for remote workers, also in remote work contexts, face-to-face communication is usually presented as the most preferred communication channel for leader-follower interactions (Smith et al., 2018), as these can better satisfy employees’ basic psychological needs. In line with leader-member exchange theory, positing that mutual trust and support is crucial for developing and maintaining high-quality relationship (Graen and Uhl-Bien, 1995), Braun et al. (2019) found that employees preferred face-to-face communication with their leaders in favor of e-mail and telephone communication, as they perceived this enhanced their mutual understanding, which was associated with higher job satisfaction. For employees who spend a lot of time working from home, video communication may compensate for the absence of face-to-face interactions. Nevertheless, research demonstrated that during the COVID-19 pandemic, long-lasting use of video calls was exhausting and caused so-called ‘zoom fatigue,’ which caused people to prefer to meet *via* other media channels (Nesher Shoshan and Wehrt, 2022).

Building on MRT (Lengel and Daft, 1988) and MST (Dennis and Valacich, 1999), we generally propose that to achieve job satisfaction in remote work contexts, frequent use of synchronous, relative rich communication channels (face-to-face communication, video calls, and telephone) remains superior to other communication channels.

*Hypothesis 3*: The positive relationship between the frequency of use of synchronous communication channels (i.e., face-to-face communication, video calls, and telephone) and job satisfaction is stronger than the relationships between the frequency of use of asynchronous communication channels (i.e., WhatsApp and e-mail) and job satisfaction.

### The moderating role of generation

Despite that generations are labeled differently, and the periods of birth years covered by these labels vary, there is consensus among academics and practitioners that generations have different norms, values, needs, and behaviors (Eisner, 2005; Anderson et al., 2017), which can also play a role in how their psychological needs are satisfied. Joshi et al. (2011) defined generational cohorts as those individuals grouped by birth years that have experienced common social and historical events during their formative years. This is of importance, particularly when comparing groups which represent individuals raised with very different technologies and lifestyles (Nicholas, 2009). Baby Boomers, stereotypically described as independent, workaholic, and disciplined, are now either retired or on the point of retirement (Eisner, 2005; Heuss et al., 2022). Gen X are considered to value greater autonomy and freedom regarding how they work; they are skeptical of authority, most likely due to the economic downturns which occurred as many of them were seeking their first job. They are not particularly fond of being micromanaged and are often in management roles themselves (Jones et al., 2019). For Baby Boomers and Gen X, education is considered necessary, and they are trying to keep up with technology. Gen Y, however, is the first generation that grew up in a world characterized by transparency, great individualism, many choices, and constant communication (Heuss et al., 2022). One of the distinctive characteristics that make Gen Y unique is that they were socialized when the development of the Internet and digital media changed the world. The literature is consistent in describing Gen Y as ‘me-oriented’ (individualistic) and as having a great desire for management support (Anderson et al., 2017), searching for meaningful jobs, work-flexibility, job satisfaction, and team collaboration in a non-hierarchical, flatter workplace (Joshi et al., 2011). Moreover, they strongly prefer to work for employers that they respect and can learn from and that support their work-life balance (Hastings, 2008).

There is some evidence that relationship-oriented leadership, characterized by inter-personal reliability, support, and trust, is valued higher among younger generations, including Gen Y, than task-oriented leadership, being more focused on personal credibility and competence (Lyons and Kuron, 2014). Younger generations also prefer to work with leaders who provide working environments that meet their individual fulfillment and ambitions rather than who focus on task and organizational success (Lyons and Kuron, 2014). Barbuto and Gottfredson (2016), for example, stressed that Gen Y prefer servant leaders who focus on employees’ developmental needs and human capital improvements. Also, according to Balda and Mora (2011), servant leadership particularly fits the needs of Gen Y, as this generation strongly values meaningful relationships with peers and supervisors, suggesting that servant leaders’ open way of communicating promotes job satisfaction. Therefore, we propose the following:

*Hypothesis 4a*: The proposed positive relationship between perceived servant leadership and job satisfaction is stronger for Gen Y compared to Baby Boomers and Gen X.

Communication frequency between leaders and followers can be associated with higher levels of interpersonal trust among remote workers (Staples, 2001). Myers and Sadaghiani (2010) noted that Gen Y seems to look for a team-based workplace culture with close contact and communication with superiors and frequent feedback to satisfy their basic psychological needs. In a similar vein, Barbuto and Gottfredson (2016) referred to a survey by Ernst and Young that found that 85% of Gen Y want frequent and fair feedback, which was higher than Gen X. A study by Gabrielova and Buchko (2021) found that Gen Y employees are entrepreneurial thinkers who like to take responsibility, demand direct and immediate feedback, expect a frequent sense of accomplishment, and have a high need for engagement and support from their manager and organization (Winter and Jackson, 2014). Since in remote work contexts, frequent communication with the supervisor is important to enhance job-satisfaction, and Gen Y values communication even more, we propose that:

*Hypothesis 4b*: The proposed positive relationship between communication frequency and job satisfaction is stronger for Gen Y than for Baby Boomers and Gen X.

As younger employees are more experienced with using lean and asynchronous communication channels, younger generations can be expected to perceive messaging as richer than older generations. In earlier research, Reisenwitz and Iyer (2009) found that Gen Y, relative to the older generations, has a higher preference for web applications and e-mail-communication and that Gen Y, who are more comfortable with technology, make use of collaborative tools, such as mobile phones, instant messaging, and social networking platforms, to connect with others and to facilitate a collective process of creative problem solving. Generally, Gen Y is more proficient at multitasking, and their preferences for communication with co-workers and peers are significantly different—and more technology-oriented—from those of Gen X. Online communication can also be an efficient way to satisfy employees’ basic psychological needs. This is in line with remote workers who have less frequent communication but perceive their communication to be of higher quality, timelier, and more efficient, which can result in job satisfaction (Fonner and Roloff, 2010). Therefore, we propose that:

*Hypothesis 4c*: The proposed positive relationship between the frequency of use of synchronous communication channels (face-to-face, video calls, telephone) and job satisfaction is weaker for Gen Y relative to Baby Boomers and Gen X.

*Hypothesis 4d*: The proposed positive relationship between the frequency of use of asynchronous communication channels (WhatsApp and e-mail) and job satisfaction is stronger for Gen Y relative to Baby Boomers and Gen X.

## Materials and methods

### Sample and procedure

This study was part of a larger, cross-sectional multi-source data collection effort on leadership and wellbeing during the COVID-19 pandemic. Data was collected in Belgium and the Netherlands through student-recruited sampling (Wheeler et al., 2014) by students enrolled in a (part-time) Master or PhD program at one of two Dutch universities. The incentive for Master student recruiters to participate was the use of the collective dataset for their thesis projects. Since the two universities had different deadlines for the thesis projects, there were two periods in which data was collected. The first data collection took place in the Netherlands between December 2020 and January 2021, while the second data collection was conducted in Belgium and the Netherlands between May and July 2021.

Since we were interested in exploring the effects of servant leadership, communication frequency, and communication channels within a remote (homeworking) context, employees who did not or hardly worked from home during the pandemic or who worked from home less during the pandemic were eliminated from the sample. In addition, in this study, we only included employees when at least two employees rated their manager’s servant leadership behavior. Cases with missing data were removed. In total, 273 employees nested in 89 managers were included in the sample. An average of 3.07 employees (range 2 to 11 employees) filled in information about their manager’s servant leadership style.

Fifty-two percent of employees in the sample were female. 53.8% of employees belonged to the millennial generation (Gen Y), while 46.2% belonged to the older generations (Baby Boomers and Gen X). The majority (80.2%) of respondents had completed a higher education degree. Student recruiters were asked to provide contact information of a diverse group of knowledge workers employed at different organizations and in different industries. Hence, respondents in our sample worked across the public and private sectors in a variety of organizations (e.g., governmental institutions, healthcare, banks, insurers, employment agencies). The majority of respondents (67.8%) worked in organizations with more than 500 employees. The mean tenure with the organization was 11.16 (SD = 10.85) years ranging from less than 1 year to 46 years. The tenure with one’s manager ranged from less than 1 year to 31 years with an average of 2.69 years (SD = 3.09). 71.43% of the employees in our sample did not work from home prior to the COVID-19 pandemic and indicated to work from home regularly or completely during the COVID-19 pandemic, while 28.57% had already worked from home prior to the pandemic and was still working (partly) from home during the pandemic.

Since the data was collected at different periods during the pandemic (December 2020 – January 2021; May – July, 2021) and within two different countries (Belgium and the Netherlands), it is important to discuss the similarities in governmental regulations during this period. In the Netherlands, in relation to the two data collection efforts, the lockdown was still in effect between December 2020 through June 5, 2021. While the Netherlands reopened after June 5, the advice (in effect from June 26, 2021) was that employees travel to the office for no more than half their workweek (RIVM, 2021). In Belgium, it was mandatory to work from home between April and July 2021. Employees were allowed to go to the office a maximum of one time per week (Belgium.be, 2021). Toward the end of the data collection (June 26 – beginning of July), the Netherlands had fewer restrictions regarding going to the office, yet only a few employees responded to the questionnaire after June 26.

### Measures

#### Servant leadership

A cross-culturally validated 18-item scale developed by Van Dierendonck et al. (2017) was used to measure servant leadership. The 18 items represent five dimensions of servant leadership (empowerment, humility, authenticity, standing back, and stewardship). The items were measured on a 6-point Likert scale ranging from (1) strongly disagree to (6) strongly agree. An example item includes ‘my manager keeps himself/herself at the background and gives credits to others’.

#### Communication frequency

Communication frequency with one’s supervisor was based on a measure presented in Webster and Wong (2008). In their study, Webster and Wong (2008) asked respondents to indicate the frequency with which they communicated with eight different media on a 7-point Likert scale (1 = never, 2 = about once a month, 3 = about once a week, 4 = about once a day, 5 = about 2–3 times a day, 6 = about 4–5 times per day, 7 = almost continuously). The sum of those eight communication media items was used to represent total communication frequency. In the present study, we adopted a similar approach, although we limited the media channels to face-to-face, telephone, e-mail, video calls, and WhatsApp. Moreover, we specifically asked employees to indicate how often they had used each of the communication channels in their communication with their direct manager during the COVID-19 pandemic. While originally, we used the 7-point Likert response scale, we recoded the variables to a 5-point scale to obtain a more equal division across response points (1 = never, 2 = about once a month, 3 = about once a week, 4 = about once a day, about 2–3 times a day, about 4–5 times a day, 5 = almost continuously). Communication frequency was calculated as the sum of the frequency with which employees communicated with their manager across all channels, with a minimum score of 5 and a maximum score of 25.

#### Frequency of use of types of communication channels

To tease out the effects of specific media channels [i.e., synchronous communication channels (face-to-face, video, and telephone) and asynchronous communication channels (e-mail, WhatsApp)], we used the recoded 5-point scales of the single items adapted from Webster and Wong (2008).

#### Job satisfaction

Job satisfaction was measured with the 4-item scale of Mossholder et al. (2005). The items were measured on a 7-point Likert scale ranging from strongly disagree to strongly agree. An example item includes ‘All in all, I am satisfied with my job’.

#### Generation

To determine generational cohort, the respondents’ age was subtracted from the year in which the data was collected. For the first data collection effort (December 2020 – January 2021), we used the year 2020. That is, since only a few respondents filled in the questionnaire in 2021, and it was only the very beginning of 2021, it made more sense to use 2020 to calculate one’s year of birth. For the second data collection effort (May – July, 2021), 2021 was used. By subtracting one’s age from the year of data collection, we could obtain a good approximation of one’s year of birth. Generational cohorts were devised based on the respondents’ year of birth. Employees born between 1981 and 2000 were grouped into the category Gen Y (coded 1). Employees born between 1946 and 1965 (Baby Boomers) and between 1966 and 1980 (Gen X) were grouped into the category older generations (coded 0).

### Confirmatory factor analyses

We conducted confirmatory factor analyses (CFAs) for the multi-dimensional scales included in our study (i.e., servant leadership, job satisfaction). The CFAs were conducted at the individual level of analysis. We first conducted a series of CFAs to examine the factorial structure of the servant leadership scale. Following Van Dierendonck et al. (2017), we compared the fit of a one-factor servant leadership model consisting of 18 items to a 5-factor model with five dimensions represented by 18 items. Both models did not have an acceptable fit (see Table 1). Despite the use of a previously validated scale, considering the results, we found it important to conduct post-hoc analyses (e.g., exploration of modification indices) and continue in an iterative, exploratory fashion.

**Table 1:** Confirmatory factor analyses.

Factor structure servant leadership^1^	*χ*2	df	*χ*2/df	CFI	TLI	RMSEA
One-factor model (18 items)	608.653	135	4.51	0.82	0.80	0.114
Five-factor model (18 items)	411.220	125	3.29	0.89	0.87	0.092
**Iterations factor structure servant leadership**						
Four-factor model (14 items)^2^	190.331	71	2.68	0.94	0.92	0.079
Higher-order four-factor model (14 items)^2^	204.318	73	2.80	0.93	0.92	0.081
**Measurement model servant leadership and job satisfaction**						
Higher-order servant leadership (14 items, 4 factors) and job satisfaction (4 items)^2^	304.709	130	2.34	0.94	0.93	0.070

*n* = 273. CFI, Comparative Fit Index; TLI, Tucker Lewis Index; RMSEA, root mean square error of approximation. ^1^This model consists of the original 18 items by Van Dierendonck et al. (2017). ^2^In this model, the authenticity dimension was not included.

According to the modification indices, one item of the humility dimension and one to two items of the authenticity dimension seemed to (cross) load on a different factor. In addition, one authenticity item had a factor loading below 0.50. When the modification indices do not offer a clear picture, EFA is more informative regarding the identification of cross-loading items than CFA (Farrell, 2010). Consequently, we performed an EFA on the 18 items. The results of the EFA showed that the three authenticity items did not load together on one factor, with one item loading on the same factor as the empowerment items. After removal of this authenticity item, the other authenticity items still did not load on the same factor. Based on this iterative process, we decided to remove the remaining two authenticity items from the scale. One humility item also did not load on the same factor as the other two items, but instead loaded together with the six empowerment items. We removed this item. After removal of this item, the remaining four factor model had an adequate fit with the data (Table 1). Considering the high correlations between the subdimensions of the four-factor model (range 0.62 to.79), following Van Dierendonck et al. (2017), we proceeded with the examination of a higher-order servant leadership model which also showed an acceptable fit with the data (Table 1).

Next, we examined a CFA consisting of the higher-order servant leadership model with four dimensions measured through 14 items and the four-item job satisfaction scale. The model showed an acceptable fit with the data, chi-square = 304.709, df = 130, chi-square/df = 2.344, CFI = 0.94, TLI = 0.93, RMSEA = 0.070. Based on the results of the CFAs, a composite score for servant leadership (average of 14 items) and job satisfaction (average of 4 items) was created.

### Analysis strategy: preliminary analyses

While our hypotheses were at the individual level of analysis, due to the nested structure of our data, it was important to determine whether having the same manager was an important source of variance in our data. We evaluated non-independence in two ways, (i) we considered whether individual employee ratings of one’s manager’s servant leadership and frequency of communication with one’s manager could be (partly) attributed to having the same manager, and (ii) we assessed whether there was significant between group variance in the outcome variable (i.e., job satisfaction).

Using the tool reported in Biemann et al. (2012), we calculated interrater reliability indices [ICC(1), ICC(2)] and computed two *r*_wg(j)_ estimates (uniform distribution and slight skew) to estimate the upper and lower bound estimate for interrater agreement on the 14-item servant leadership measure. The results showed an upper bound *r*_wg(14)_ of 0.92, SD = 0.23 (uniform distribution) and a lower bound *r*_wg(14)_ of 0.87, SD = 0.26 (slight skew). Based on the lower bound *r*_wg(14)_ estimates (slight skew), 89.89% of groups had strong (0.71–0.90) to very strong agreement (0.91–1.00). For the upper bound, 94.38% of groups had strong to very strong agreement. The ICC(1) is 0.16 and the ICC(2) is 0.37, F ratio = 1.60 *p* < 0.01. The “F ratio is the result of an ANOVA-based significance test of between-group differences and indicates the statistical significance of group membership” (Biemann et al., 2012, p. 78).

In addition to servant leadership ratings, we also asked employees to indicate the frequency of communication with their manager through five different media channels. While the items focused on individual communication with one’s manager, it might be that how often and in what way managers communicate with their employees is (partly) shared among employees. We, therefore, examined the ICC(1), ICC(2) and *r*_wg_ (uniform distribution and moderate skew) for the single-item communication frequency measures by means of Biemann et al.’s tool which also allows for the calculation of these indices for single-item measures. In Table 2 (format adapted from Biemann et al., 2012), we present the results for the single-item measures for the uniform and measure-specific (moderate skew) indices. Since the composite score is based on the sum of these single-item constructs, similar levels of agreement on the composite score are assumed. Although the significant F-tests and ICC(1) and ICC(2) values warrant aggregation, even when considering the upper bound *r*_wg_ indices (uniform distribution), for several communication channels there are a considerable number of groups with lack of, weak or moderate agreement. This entails that within numerous groups, the aggregated score may not represent any of the raters’ perspectives adequately (*cf.* Klein and Kozlowski, 2000). Nevertheless, it is important to not simply ignore potential group-level effects, as “researchers may draw erroneous conclusions (due to biased standard errors) when unit membership is a known source of variance but is excluded from statistical analyses” (Biemann et al., 2012, p. 72).

**Table 2:** Results aggregation analyses - frequency communication channels.

	*r*_wg.uniform_		*r*_wg.moderate skew_					
Measure	Mean	SD	Variance	Mean	SD	F ratio	ICC(1)	ICC(2)
Face-to-face communication	0.84	0.24	0.90	0.72	0.32	3.12***	0.41	0.68
Telephone communication	0.68	0.33	0.90	0.50	0.37	2.23***	0.29	0.55
E-mail communication	0.71	0.31	0.90	0.53	0.38	2.28***	0.30	0.56
Video communication	0.72	0.30	0.90	0.53	0.38	1.99***	0.24	0.50
WhatsApp communication	0.64	0.31	0.90	0.41	0.35	2.27***	0.29	0.56

SD, standard deviation of *r*_wg_ values. Variance estimations for moderate skew were based on calculation values from LeBreton and Senter (2008, p. 832) as used in the computational tool of Biemann et al. (2012). ***p < 0.001.

To assess the extent of non-independence in job satisfaction, we calculated ICC(1) by dividing the interclass variance by the sum of the interclass variance and intraclass variance (ICC(1) = 0.062/(0.062 + 1.104) = 0.053) based on the null model with restricted maximum likelihood estimation (Heck et al., 2014). The ICC(1) value was at the cut-off point of 0.05 for conducting multilevel analyses (González-Romá and Hernández, 2017). Although it has been suggested that non-independence of 0.05 or lower in the outcome variable does not require multi-level analyses, the interrater agreement (*r*_wg_-based indices) and interrater reliability (ICC1 and ICC2) indices for servant leadership and the communication frequency variables point to potential group-level effects that should not be neglected. Consequently, we decided to consider both the individual level and the group level components of servant leadership and the communication variables in our analysis (i.e., we included the predictor variables at both the first and second level of analysis). By taking this approach, we had the opportunity to tease out unique estimates for within and between-group coefficients.

### Data analysis strategy: hypothesis testing

We conducted multi-level analyses through MIXED MODELS Linear with restricted maximum likelihood estimation in SPSS version 28. Since ‘communication frequency’ was constructed as the sum of the frequency on the individual communication channels it was not possible to include these variables together in one analysis (there would be perfect collinearity with one individual communication channel variable and the composite communication frequency score). Hence, we tested Hypotheses 1 and 2 (Model 1a) simultaneously and Hypotheses 1 and 3 (Model 2a) simultaneously. In both models, the level 2 predictor variables were grand mean centered, while the level 1 predictors were centered within cluster (CWC) also known as group-mean centered (Enders and Tofighi, 2007). This type of centering at level 1 and level 2 offers the opportunity to tease out within and between level effects (Enders and Tofighi, 2007). Generation, the level 1 moderator variable, was group-mean centered (CWC) to accommodate interpretation of interaction terms at level 1 (Enders and Tofighi, 2007).

**Figure 1:**
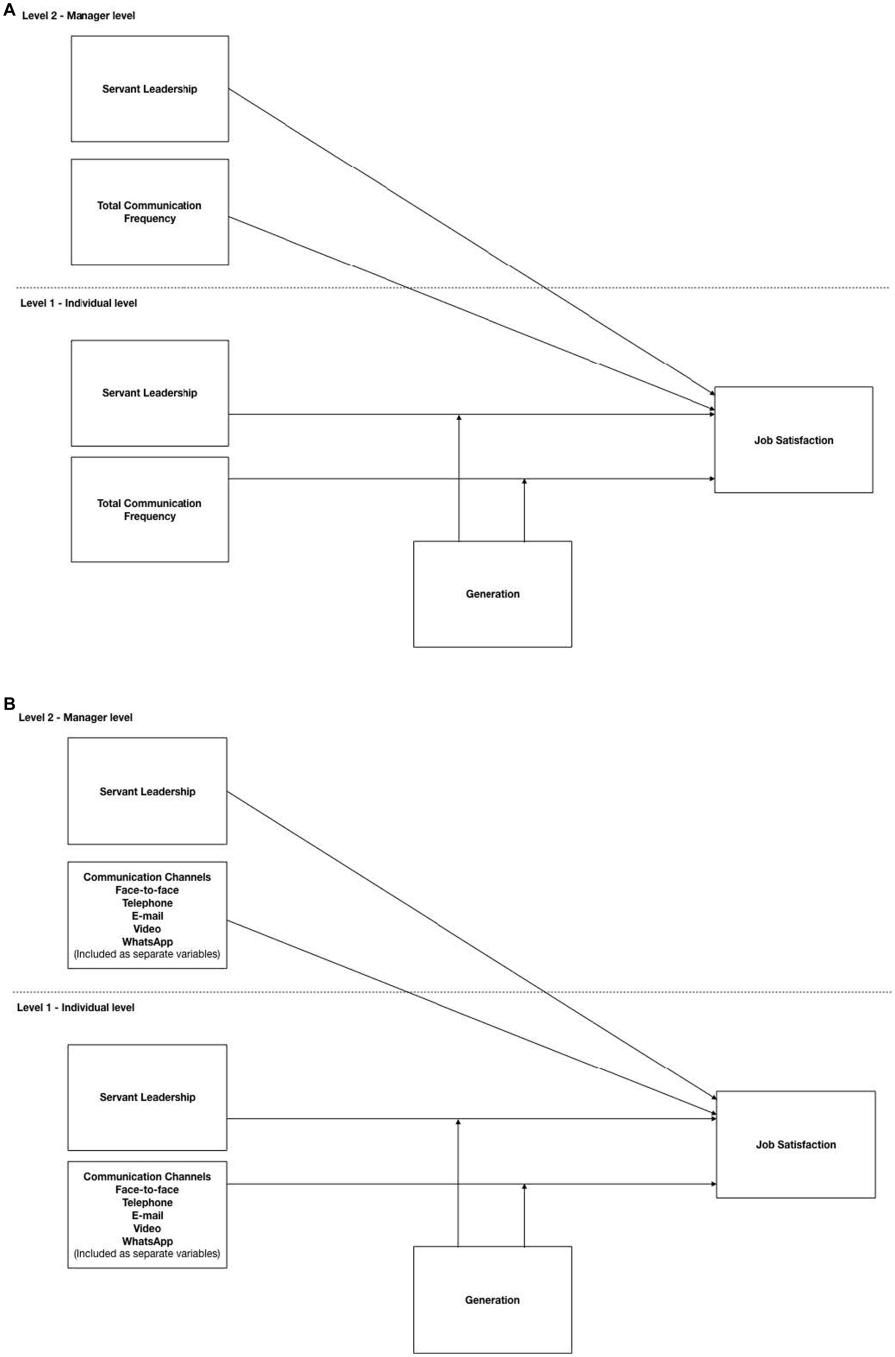
Proposed empirical models.

To examine whether generation moderated the relationship between level 1 predictor variables and job satisfaction (Hypotheses 4a–4d), the interaction terms were included to form Model 1b (Hypotheses 4a, 4b) and Model 2b (Hypotheses 4a, 4c, 4d). The empirical models used to test the hypotheses are presented in Figure 1. The model in Panel A captures Hypotheses 1, 2, 4a and 4b, while the model in Panel B displays Hypotheses 1, 3, 4a, 4c, 4d.

As working with one’s manager for less than 1 year, particularly when this was (partly) during a lockdown, may not offer sufficient time to develop an understanding of one’s manager’s leadership style or an awareness of communication norms, we also conducted the analyses on a sample excluding employees who had less than 1-year tenure with their manager.^1^ Furthermore, while we were interested in comparing the experiences of the younger generation (Gen Y) to older generations (Gen X and baby boomers), since there might still be important differences between Gen X and baby boomers, we also conducted the analyses among a sample limited to Gen Y and Gen X respondents only.^2^ Due to the relatively small percentage of baby boomers in the total dataset (11.63%), we did not conduct analyses comparing only Gen Y and baby boomers. The outcome of the hypothesis tests remained the same; therefore, we present the results including the full sample of 273 employees.

## Results

In Table 3, the descriptive statistics (means, SDs, and reliability coefficients) and bivariate correlations among the variables are presented. Servant leadership was significantly positively correlated with job satisfaction (*r* = 0.30, *p* < 0.01). Of the communication variables, only e-mail communication was significantly correlated with job satisfaction (*r* = 0.13, *p* < 0.05). While frequency of use of the different types of communication channels (face-to-face, telephone, e-mail, videoconferencing, WhatsApp) were generally significantly positively correlated with each other (*r* ranges from 0.18 to 0.54, *p* < 0.01), face-to-face and WhatsApp communication were not significantly correlated (*r* = 0.09, *p* > 0.05).

**Table 3:** Descriptive statistics.

Variable	Mean	SD	1	2	3	4	5	6	7	8	9
1. Servant Leadership	4.72	0.71	(0.92)								
2. Communication Frequency	12.59	3.28	0.11	.							
3. Face-to-face Communication	1.61	0.80	0.05	0.48**	.						
4. Telephone Communication	2.70	1.01	0.02	0.77**	0.33**	.					
5. E-mail Communication	3.23	0.99	0.10	0.78**	0.18**	0.54**	.				
6. Videoconferencing	2.68	0.89	0.11	0.68**	0.20**	0.38**	0.43**	.			
7. WhatsApp Communication	2.38	1.09	0.10	0.69**	0.09	0.34**	0.46**	0.34**	.		
8. Generation	0.54	0.50	0.06	0.04	0.04	−0.00	−0.01	0.03	0.07	.	
9. Job Satisfaction	5.57	1.08	0.30**	0.06	0.01	−0.02	0.13*	0.02	0.07	−0.01	(0.90)

*n* = 273, *p < 0.05, **p < 0.01. Cronbach’s alphas are displayed on the diagonal in parentheses. Generation is coded 1 (Gen Y) and 0 (Gen X and Baby Boomers).

According to Hypothesis 1, there is a positive relationship between servant leadership and job satisfaction. The results of Model 1a and Model 2a show that there is a positive relationship between individual-level servant leadership and job satisfaction (estimate = 0.55, *p* < 0.05, and estimate = 0.53, *p* < 0.05 for Model 1a and 2a respectively). Moreover, group-level servant leadership and job satisfaction are significantly positively related (estimate = 0.36, *p* < 0.05, and estimate = 0.37, *p* < 0.05 for Model 1a and 2a respectively; see Tables 4, 5). Hypothesis 2 proposed a positive relationship between communication frequency with one’s supervisor and job satisfaction. According to the results (Table 4), there is no significant relationship between group-level communication frequency and job satisfaction (estimate = 0.02, *p* > 0.05), nor for individual-level communication frequency and job satisfaction (estimate = −0.01, *p* > 0.05). Hence, Hypothesis 2 is not supported. Fonner and Roloff (2010) found that fewer exchanges of information was related to higher job satisfaction among high-intensity teleworkers compared to office workers. Hence, it might be that among our sample (which consisted of intense homeworkers due to government regulations during the pandemic), the frequency of communication with one’s manager might not have a linear effect, but the positive effect may plateau at a certain level. Consequently, we conducted post-hoc analyses examining quadratic and cubic relationships between total communication frequency and job satisfaction. There was no support for curvilinear effects.

**Table 4:** Results hypothesis tests (hypotheses 1, 2, 4a, 4b).

	Model 1a			Model 1b		
	Coef.	SE	*t*	Coef.	SE	*t*
**Fixed effects**						
Intercept	5.573	0.069	80.669***	5.573	0.069	80.318***
**Level 1**						
Servant leadership	0.55	0.114	4.830***	0.55	0.117	4.663***
Communication frequency	−0.01	0.027	−0.390	−0.01	0.027	−0.376
Generation	−0.20	0.162	−1.205	−0.20	0.163	−1.214
Servant leadership * generation				0.02	0.341	0.044
Communication frequency * generation				−0.03	0.076	−0.440
**Level 2**						
Servant leadership	0.36	0.144	2.490*	0.36	0.146	2.459*
Communication frequency	0.02	0.029	0.833	0.03	0.029	0.857

*N* = 273, *k* = 89. Generation (1 = Gen Y; 0 = Gen X and Baby Boomers). *p < 0.05, **p < 0.01, ***p < 0.001.

**Table 5:** Results hypothesis tests (hypotheses 1, 3, 4a, 4c, 4d).

	Model 2a			Model 2b		
	Coef.	SE	*t*	Coef.	SE	*t*
**Fixed effects**						
Intercept	5.566	0.067	82.736***	5.571	0.069	80.226***
**Level 1**						
Servant leadership	0.53	0.117	4.564***	0.51	0.120	4.263***
Face-to-face communication	0.08	0.124	0.652	0.08	0.126	0.666
Telephone communication	−0.12	0.112	−1.099	−0.14	0.113	−1.216
E-mail communication	0.03	0.120	0.280	0.03	0.122	0.220
Videoconferencing	0.03	0.111	0.296	0.04	0.112	0.335
WhatsApp communication	−0.01	0.092	−0.123	−0.01	0.093	−0.066
Generation	−0.19	0.166	−1.164	−0.22	0.169	−1.300
Servant leadership * generation				−0.04	0.353	−0.102
Face-to-face * generation				−0.41	0.410	−0.987
Telephone * generation				−0.31	0.286	−1.065
E-mail * generation				0.31	0.328	0.953
Video * generation				−0.10	0.319	−0.322
WhatsApp * generation				0.13	0.268	0.477
**Level 2**						
Servant leadership	0.37	0.143	2.557*	0.36	0.147	2.453*
Face-to-face communication	−0.06	0.118	−0.506	−0.05	0.121	−0.431
Telephone communication	−0.07	0.115	−0.569	−0.07	0.118	−0.623
E-mail communication	0.26	0.125	2.093*	0.24	0.131	1.799^†^
Videoconferencing	−0.11	0.122	−0.896	−0.11	0.125	−0.883
WhatsApp communication	0.03	0.099	0.282	0.05	0.105	0.500

*N* = 273, *k* = 89. Generation (1 = Gen Y; 0 = Gen X and Baby Boomers). ^†^*p* < 0.10, *p < 0.05, **p < 0.01, ***p < 0.001.

According to Hypothesis 3, there would be a positive relationship between all communication channels and job satisfaction, while the relationship between synchronous communication channels (face-to face, video, telephone) and job satisfaction was expected to be stronger than the relationship between asynchronous (e-mail, WhatsApp) communication and job satisfaction. The results do not support Hypothesis 3. While there are no significant relationships between the different communication channels and job satisfaction at level 1 (see Table 5), there is a significant positive relationship between e-mail communication at level 2 and job satisfaction (estimate = 0.26, *p* < 0.05). This is contrary to the expectation that synchronous communication channels would have the strongest relationship with job satisfaction.

According to Hypothesis 4a, the relationship between servant leadership and job satisfaction would be moderated by generation. There was no support for the moderating effect of generation (estimate = 0.02, *p* > 0.05, Model 1b; estimate = −0.04, *p* > 0.05, Model 2b).

According to Hypothesis 4b, the relationship between communication frequency and job satisfaction would be stronger for Gen Y. Considering the non-significant interaction effect (estimate = −0.03, *p* > 0.05), Hypothesis 4b is not supported.

Hypothesis 4c proposed that the positive relationship between synchronous communication channels and job satisfaction would be weaker for Gen Y, while Hypothesis 4d suggested that the positive relationship between asynchronous communication channels and job satisfaction would be stronger for Gen Y. There were no significant interaction effects (estimate = −0.41, *p* > 0.05, estimate = −0.31, *p* > 0.05, estimate = 0.31, *p* = 0.10, estimate = −0.10, *p* > 05, and estimate = 0.13, *p* > 0.05 for the level 1 interactions between face-to-face, telephone, e-mail, video conferencing and WhatsApp communication and generation respectively), and therefore these hypotheses were not supported (Table 5).

## Discussion and conclusions

Particularly in remote work contexts, examining contextual autonomy factors in job satisfaction is important, as remote working can reduce employees’ motivation and enhance turnover intention (Charalampous et al., 2019; Zöllner and Sulíková, 2021). In this section, we summarize our study’s results and reflect on them in light of existing theory and research. Moreover, we discuss the study’s limitations and avenues for future research and implications for management practice.

### The relationship between servant leadership and job satisfaction

In line with expectations, we found a positive relationship between servant leadership and job satisfaction. When leaders were perceived as enacting servant leadership behaviors, their followers reported more job satisfaction, possibly because their leaders’ behaviors satisfied their basic psychological needs for autonomy, relatedness, and competence (Deci and Ryan, 2000; Van Dierendonck et al., 2014). Servant leaders can shape work environments for their followers that enable building trustful relationships and that can enhance individuals’ autonomous motivation and job satisfaction. More specifically, by enhancing autonomy, building working communities that foster belongingness, and allocating interesting tasks that allow employees to use their capacities and develop these, leaders can sustain employees’ proactive work behavior, also in remote work contexts (Coun et al., 2022). This also relates to the outcomes of the meta-analysis by Gajendran and Harrison (2007) that indicated that teleworkers are more satisfied with their jobs when they can embrace the potential benefits and advantages of remote working, such as more time-spatial flexibility, job autonomy, and improved supervisory relationships. Although previous studies did not always consider a multilevel design, our finding is in line with other studies (Chan and Mak, 2014; Donia et al., 2016) that showed a positive relationship between servant leadership and job satisfaction. In addition, many studies looking into servant leadership and job satisfaction during the COVID-19 pandemic were conducted in the educational and healthcare context (Qiu and Zhang, 2022; Turner, 2022). However, our study broadened the focus by including knowledge workers in a wide range of both public and private sectors that had to work remotely.

### The relationship between communication frequency and job satisfaction

We expected that particularly within the context of remote working during the COVID-19 pandemic frequent leader-follower communication was important as this can contribute to employees’ perceptions of support to work autonomously, to build professional and social relationships, and to develop professionally, which enables them to deal with stress in times of crisis and to prevent them from professional isolation (Marshall et al., 2007; Golden et al., 2008), enhancing autonomous motivation and, subsequently, job satisfaction (Rhoades and Eisenberger, 2002). This expectation, however, was not supported by our data. Perhaps at the time of research, the employees in our sample were already used to the new remote work situation during the COVID-19 pandemic and did not feel that they needed frequent communication to interact with their supervisor to be autonomously motivated to do their work remotely. Martin et al. (2022), for example, found that in a context of perceived information overload, more frequent communication was negatively related to job satisfaction. Also before the pandemic, Fonner and Roloff (2010), Leonardi and Barley (2010), and Ter Hoeven et al. (2016) found that employees intentionally reduced the interaction frequency with the shop-floor level to be able to work more uninterruptedly and to focus and concentrate on their own work (Leonardi and Barley, 2010), perhaps to achieve more work-related flow (Peters and Wildenbeest, 2010; Peters et al., 2014) or to have more time for nonwork activities, such as ‘home schooling’ for their children due to schools being closed to prevent the COVID-19 virus from spreading. Also, in our study, lower communication frequencies in high-intensity remote work contexts may have contributed to lower stress from meetings and interruptions, less office politics, and, consequently, less pondering about work after work hours, resulting in less work-life conflict (Fonner and Roloff, 2010). The lack of significance in our analysis might indicate that there is not a particular pattern that relates higher or lower levels of communication intensity with job satisfaction. This resonates with Boell et al. (2016, p. 128) who pointed out the importance of looking into “the complexity and mutual dependencies of situated work activities” to understand remote work practices. In addition, they suggested that the diversity of work activities in which knowledge workers engage and the different IT-tools they use, can determine the need for and appreciation of interaction during remote working.

### The relationship between the communication channel frequency and job satisfaction

Surprisingly, we only found a significant direct positive relationship between e-mail communication frequency and job satisfaction. The other communication channel frequencies of individual video calls (e.g., *via* Teams, Skype, or Zoom), individual telephone calls, and WhatsApp, however, were not significant. We expected that frequent face-to-face communication, or in remote work contexts frequent video calls, which are usually presented as the most channel rich in terms of information availability and immediacy of feedback and the opportunity to send nonverbal cues and personal focus, would be the preferred communication channels for leader-follower interactions (Smith et al., 2018), and, for those reasons, would have had positive relationships with job satisfaction. However, frequent means of synchronous and media rich communication did not relate to higher levels of job satisfaction. This may imply that the experienced quality of the communication in remote work contexts does not depend on the quantity of rich and synchronous communication (Fonner and Roloff, 2010). Since face-to-face communication during the COVID-19 pandemic was reduced, even in times when national lockdowns were more relaxed, one would expect that frequent video calls or making telephone calls would be alternative synchronous communication channels that have the potential to compensate for the loss of face-to-face communication. However, also higher frequencies of these types of synchronous communication did not contribute to job satisfaction. Based on the literature, the finding regarding video calls could be attributed to ‘zoom fatigue’ (Nesher Shoshan and Wehrt, 2022). More specifically, zoom fatigue can be explained by more subjective processes rather than objective processes related to media richness and synchronicity. Subjective processes rather relate to the specific cultural-symbolic meanings of communication channels that can affect employee attitudes and reactions and enhance channel-usage related strain (Nesher Shoshan and Wehrt, 2022). In addition to the loss of richness of social cues and the difficulties with signaling these, video conferencing and the technical problems that occurred might remind them of the normal face-to-face communication that was lost during the pandemic, which depleted employees’ energy resources (Nesher Shoshan and Wehrt, 2022). Additionally, telephone communication may not compensate for missing out face-to-face communication.

Apparently, however, in remote work contexts, frequent e-mail communication as a characteristic of the leader was perceived by employees as contributing to job satisfaction, and strikingly also even more than the other types of a-synchronic communication, such as WhatsApp. Probably e-mail communication allows followers to have ongoing contact with their supervisor and to exchange more information with their supervisor in an a-synchronic way. On the one hand, information exchange is an important part of empowering leadership, also characterizing servant leadership (Van Dierendonck et al., 2014), and can foster employee empowerment, which enables employees’ work proactivity (Coun et al., 2022), and, therefore, may contribute to job satisfaction. On the other hand, remote workers may increasingly search for efficient ways of communication and for gaining more job autonomy and flexibility, to concentrate on work and/or nonwork activities (Gratton, 2023). E-mail communication may be a channel that allows them to be better able to control when and how to respond to the leader’s information (*cf.* Fonner and Roloff, 2010), meanwhile offering the advantage of continuity in leader-follower conversations (Smith et al., 2018). Perhaps individual telephone calls can be richer in terms of receiving direct responses from the supervisor, but smaller portions of information can be exchanged. Furthermore, WhatsApp may be a more flexible, but less rich medium. Or this more synchronous channel can give employees pressure to respond faster (Smith et al., 2018) or this might be associated with more informal, supplemental communication (Darics, 2020).

### The moderating role of generation

In contrast to our hypotheses, we did not find any significant moderating influence of generation. Although we expected that the younger generation (Gen Y) would benefit more from servant leadership than the older generations (Baby Boomers and Gen X), the results did not support that Gen Y would be in need of more servant leadership to be satisfied with their work. Nor did we find a stronger relationship between communication frequency and job satisfaction among the younger generation compared to the older generations, although Gen Y was expected to value more direct communication and their managers to be mentors at work (Winter and Jackson, 2014). Regardless of generation, the preferred communication frequency might be dependent on the complexity and interdependencies across workers in remote work settings. Strikingly, however the frequency of communication did not a play a different role regarding different communication channels. Possibly, all employees in our study were experienced in using different communication channels which can explain the non-significant moderation effect of generation. In fact, the pandemic may have reduced differences in experience with the use of different communication channels. In view of the ‘age-period-cohort confound’ (Rhodes, 1983), our study may be a result of a period effect. In future research, to disentangle this confound, a longitudinal design is essential.

### General conclusion

Our study contributed to the conversation on leadership style and wellbeing (Inceoglu et al., 2018) by showing that servant leadership in remote work contexts, often characterized by uncertainty and complexity, plays an important role in fostering employees’ job satisfaction (Fernandez and Shaw, 2020; Banks et al., 2022; Tigre et al., 2022). This holds true for all generations. Servant leadership is a multi-faceted leadership style that focuses on task clarification and building internal and external relationships and has the potential to fulfil employees’ psychological needs in terms of autonomy, competence, and belongingness (Van Dierendonck, 2011) which motivates them for their work resulting in higher levels of job satisfaction. This, however, does not imply that leaders need to interact frequently with their followers. In line with previous studies, our study did not find support for a significant relationship between communication frequency and job satisfaction in general. Likely, the type of work and the personal needs of individuals and teams determine the ideal leader-follower interaction frequency. Yet, by simultaneously looking to leadership and the use of communication media, we found that at the leader level, also high intensities of e-mail communication can be associated with higher levels of job satisfaction. This may imply that besides human-centric leadership styles, asynchronous communication is becoming increasingly important in remote work contexts, as this allows employees to be more autonomous and flexible and at the same time able to share knowledge with their teammates. Attention and interaction with the supervisor remain important, but it may depend on the type of work (Boell et al., 2016). Besides the indicators of media richness (Lengel and Daft, 1988) and media synchronicity (Dennis and Valacich, 1999) also the social symbolic value of communication might affect job satisfaction. In our study, empowering employees by sharing information *via* e-mail reflects leaders to involve and trust employees to do their work autonomously. Paradoxically, leaders need to signal that they are supporting their teams and individual employees but at the same time they should give employees autonomy and flexibility to do their work independently and collaborate with others remotely. This fits with the trend indicated by Gratton (2023) that people after the COVID-19 pandemic prefer to combine work with other activities outside the work domain. This holds true for employees regardless of generation.

## Limitations and future research

The present study was subject to various limitations. First, the present study revealed the paradox of leaders both signalling support and autonomy. It is not clear how leaders toggle between these two poles to keep employees satisfied. Future research could explore how managers in hybrid work contexts deal with ambiguous and complex situations and choose the right media to communicate with their employees (Bergum et al., 2023).

Second, we investigated the role of servant leadership in job satisfaction of employees nested within managers. In hybrid work contexts, however, employees increasingly must collaborate with others outside their team or workgroup and their organization. Future research could examine how servant leaders take up their role to avoid siloing and act as bridge between teams and organizations to stimulate collaboration and open innovation (Edelbroek et al., 2019).

Third, although no differences across generations were found regarding the relationship between servant leadership and job satisfaction, possibly, servant leaders need to display different behaviors depending on the experience of younger and older generations in the organization. In hybrid work contexts, the role of servant leaders may be increasingly important, particularly during the onboarding processes (Bauer and Erdogan, 2011), focusing on compliance with rules and regulations, clarification of tasks, learning about organizational culture and making connections with others inside and outside the organization. The importance of onboarding in remote work contexts stresses the need for servant leaders to differentiate their behaviors, communication frequency and communication channels. Future research could focus on the role of servant leaders in relation to generations and age differences during onboarding processes.

Fourth, the cross-sectional nature of our data did not allow us to examine causality. Fifth, while overall the r_wg(j)_ indices warranted aggregation of servant leadership, some groups of employees included in our sample lacked agreement or had low agreement on the servant leadership rating of their manager. Moreover, while within group agreement [r_wg(j)_] and the significant F-test of the ICC indices justified our consideration of servant leadership at both the individual and leader-level, the ICC(1) and ICC(2) values for servant leadership in our study were lower than those in other studies which included servant leadership at the group level (Hunter et al., 2013; Harju et al., 2018). While the ICC(2) value was likely lower due to the low number of average raters per group, the ICC(1) values do not vary across group size (Klein and Kozlowski, 2000). Although ICC(1) values of 0.10 to 0.25 indicate a medium effect and justify aggregation (LeBreton and Senter, 2008), it is surprising that the ICC(1) values in the present study were lower in comparison to other studies measuring servant leadership.

Sixth, based on post-hoc exploratory analyses of the servant leadership measure, we had to remove all three items belonging to the subdimension authenticity. This subdimension included items such as ‘my manager shows his/her true feelings towards his/her staff’. While we used a previously cross-culturally validated measure of servant leadership (Van Dierendonck et al., 2017), it might be that the COVID-19 pandemic and pursuant social distancing and work-from-home regulations stipulated by the government did not offer sufficient opportunity for managers to show authenticity across employees in the sample, which led to inconsistencies and loadings with items from other servant leadership dimensions. That is, not all managers may have felt comfortable sharing/showing feelings in a hybrid work environment. Hence, it is interesting to further explore the universal applicability of servant leadership dimensions in crisis contexts.

Seventh, although we did not consider changes in communication frequency across different communication channels over time, we presume face-to-face communication decreased due to lockdowns and governmental regulations stipulating employees to work at home when the situation allowed. However, it might be that for our particular sample of knowledge workers, face-to-face interaction and other types of interaction with one’s supervisor was relatively low before lockdowns and social distancing measures due to the relatively high levels of autonomy of knowledge workers. Hence, in future research it is interesting to examine whether communication frequency differentially affects job satisfaction of knowledge versus blue-collar workers and whether, if such differential effects exist, this can be explained by the autonomy one has in one’s job.

Finally, while it is not uncommon to examine communication frequency based on frequency with different types of communication channels, our study could have benefited from an approach which distinguishes between communication initiated by the employee versus that initiated by one’s supervisor (Kacmar et al., 2003), as the frequency with which one’s manager initiates interaction, particularly in unprecedented situations such as the COVID-19 pandemic, may play a role in an employee’s attitudinal responses in the workplace. In addition, future research could also benefit from considering the role of the length of communication across communication channels. Exchanging multiple text messages a day could be experienced differently from engaging in a 3-h videocall, as the latter may lead to ‘zoom-fatigue’ (Nesher Shoshan and Wehrt, 2022). On the other hand, exchanging text messages several times throughout a day could be experienced as distracting by some employees. Furthermore, in the present study, we did not consider how employees appraised the communication exchanges, which could be relevant for how satisfied they are with the work environment and the support received. In future research, the appraisal of the communication exchanges could also be considered.

## Managerial implications

Aside from its theoretical contributions, our study provides guidance to practitioners in the field. Especially in light of organizations considering the adoption of a new hybrid model of working (Gratton, 2021), our study strongly advocates the use of servant leadership behaviors to foster job satisfaction in employees, in remote work contexts.

Rather than focussing on frequent communication, managers should focus on the need for synchronous versus asynchronous communication which may depend on the tasks at hand and personal needs of the employee and teams (Boell et al., 2016).

Servant leaders can help remote workers to adapt to their changing work context but at the same time should leave sufficient autonomy for them to shape their work in line with their preferences. This demands management training and coaching as well. Asynchronous communication can both empower employees and offer them the flexibility they need. As post-pandemic organizations are implementing hybrid remote work policies, communication expectations will become even more important as people navigate multiple working environments.

## Data availability statement

The datasets presented in this article are not readily available because respondents consented to the sharing of their data for authentication purposes only. Requests to access the datasets should be directed to the authors.

## Ethics statement

Ethical review and approval was not required for the study on human participants in accordance with the local legislation and institutional requirements. The participants were provided a detailed written explanation of the data management procedures. To be able to match employees to managers, personal identifying information (e.g., e-mail addresses) was collected. Codes were used in the matching process. After the matching process was completed, personal identifying information, such as e-mail addresses, used in the matching process, was removed from the dataset and stored separately in an encrypted environment; only the researchers directly involved in this study have access to this information. Respondents were ensured that their personal answers would not be shared with their managers or coworkers. Student recruiters involved in the data collection received a pseudonymized dataset for their own research projects. Furthermore, participants were ensured that personal identifying information would not be presented in reports or publications. Participants were informed about data storage procedures. Moreover, participants were informed that participation was voluntary and they could withdraw at any time. The studies were conducted in accordance with the local legislation and institutional requirements. The participants provided their written informed consent to participate in this study.

## Author contributions

All authors listed have made a substantial, direct, and intellectual contribution to the work, and approved it for publication.

## Conflict of interest

The authors declare that the research was conducted in the absence of any commercial or financial relationships that could be construed as a potential conflict of interest.

## Publisher’s note

All claims expressed in this article are solely those of the authors and do not necessarily represent those of their affiliated organizations, or those of the publisher, the editors and the reviewers. Any product that may be evaluated in this article, or claim that may be made by its manufacturer, is not guaranteed or endorsed by the publisher.

## Footnotes

^1^Eighteen employees had tenure less than 1 year; after removing these respondents, there were 7 cases where only one employee filled in information about one’s manager, these cases were also removed. The sensitivity analyses were therefore performed among 248 employees nested in 82 managers.

^2^Thirty-one employees belonged to the Baby Boom generation; after removing these respondents, there were 12 cases where only one employee filled in information about one’s manager, these cases were also removed. The sensitivity analyses were therefore performed among 230 employees nested in 77 managers.
